# Supplementary material for: The effects of free trade agreements on the stock market: Evidence from Vietnam
Source: PLoS One. 2024 Feb 29;19(2):e0294456. doi: 10.1371/journal.pone.0294456 (PMC10903835; doi:10.1371/journal.pone.0294456)
Supplement: Attachment — Submitted filename: Comments.docx [file pone.0294456.s001.docx]

Comments

**Point 1**: Introduction: Further explanation is needed on the impact of EVFTA on the Vietnam stock market and to provide intuitive data support for this impact. In addition, it is necessary to explain the uniqueness of the Vietnam stock market as an emerging market.

**Point 2**: Literature Review: ①The length is too long, there are many literature listed, and it is not closely combined with the viewpoints proposed in the article, such as the impact on various returns and risks. ②The author needs to provide a brief summary of existing literature. ③I suggest adding authoritative references appropriately and reflecting the forefront of research.

**Point 3**: Methodology: The author needs to construct a mechanism diagram to reflect the unique impact of EVFTA on emerging markets (Vietnam stock market), and further structure the logical thinking of the paper.

**Point 4**: Data and Empirical Results: ①The author needs to check and confirm that Event 3 in Table 6 corresponds to Food & Drugs Retailers? It is mentioned in Early and Delayed Reaction Analysis. What I see is that Event 10 corresponds to Food&Drugs Retailers, and Event 3 corresponds to Gas, Water & Multiutilities. ②In the Risk Structure Analysis section, the author should further reveal the reasons for the short-term changes in systematic risk after Event 5. ③Are Figures 1 and 2 repeated twice? The display effect of the two figures are not clear. ④Besides different industries, do the nature of enterprises or the market value of companies also have different effects?

**Point 5**: Conclusion: Introduction mentions the significance of studying the impact of EVFTA on the Vietnam stock market, which mentioned “It ... which will be useful to investors and policymakers in general.”The conclusions now only involve a summary of the empirical results. Policy implications need to be added to echo the previous views.
