# Supplementary material for: The effects of free trade agreements on the stock market: Evidence from Vietnam
Source: PLoS One. 2024 Feb 29;19(2):e0294456. doi: 10.1371/journal.pone.0294456 (PMC10903835; doi:10.1371/journal.pone.0294456)
Supplement: Attachment — Submitted filename: Responses to Reviewers.docx [file pone.0294456.s002.docx]

**Responses to Reviewers’ Comments**

We thank the Editors and Reviewers for your valuable comments and recommendations, which help significantly improve our study. We attended to all your comments and would like to provide details on how we addressed them.

**Reviewer #1:** This paper discusses the effects of Free Trade Agreements on the Vietnam stock market. Manuscripts describe, in part, technically sound scientific research and provide data to support conclusions. It is rich in content, but there are still some problems.

1. ***Introduction: Further explanation is needed on the impact of EVFTA on the Vietnam stock market and to provide intuitive data support for this impact. In addition, it is necessary to explain the uniqueness of the Vietnam stock market as an emerging market.***

We would like to thank the editor and reviewers for their valuable feedback. In response to the reviewers' suggestions, we have made significant revisions to the manuscript. We have restructured the introduction section as follows: (1) Providing context on Vietnam and its Free Trade Agreements (FTAs), (2) Discussing the specific context of Vietnam in relation to the EU-Vietnam Free Trade Agreement (EVFTA) and its impact on the country, (3) Highlighting the gap in existing research and outlining the contributions of our study, (4) Explaining the rationale for examining the EVFTA and its implications for the Vietnam Stock market, and (5) Presenting an overview of the structure of our research.

Additionally, we added a paragraph to provide detailed explanations about the Vietnam stock market as well as the uniqueness of the market (paragraph 2 on page 5). We also explain the need to test the effect of EVFTA on the Vietnam stock market (Paragraphs 1, 2 and 3 on page 5).

2. ***Literature Review: ① The length is too long, there are many literature listed, and it is not closely combined with the viewpoints proposed in the article, such as the impact on various returns and risks. ② The author needs to provide a brief summary of existing literature. ③ I suggest adding authoritative references appropriately and reflecting the forefront of research.***

We have revised and restructured our literature and hypotheses development section by summarizing existing literature and providing empirical literature with targeted markets. We have provided discussions on the impact of FTA news and events on the stock market return, and on how the EVFTA news and events provide different impacts on various industry sectors in the Vietnam stock market. We have consolidated the discussion on the impact of FTAs on the market risk across industries and countries. In so doing, we have included the recent related literature, and thus highlighted the research gap from the existing literature and formulated the three testable hypotheses as follows.

H1: EVFTA-related events significantly affect the Vietnam stock market.

H2: The effects of EVFTA-related events vary across Vietnamese sectors.

H3: EVFTA announcements have different impacts on systematic risk across the sectors in Vietnam.

3. ***Methodology: The author needs to construct a mechanism diagram to reflect the unique impact of EVFTA on emerging markets (Vietnam stock market), and further structure the logical thinking of the paper.***

We have added Figure 1 on page 40 to show the mechanism diagram to show the impact of EVFTA on the Vietnam stock market.

4. ***Data and Empirical Results: ①The author needs to check and confirm that Event 3 in Table 6 corresponds to Food & Drugs Retailers? It is mentioned in Early and Delayed Reaction Analysis. What I see is that Event 10 corresponds to Food&Drugs Retailers, and Event 3 corresponds to Gas, Water & Multiutilities. ②In the Risk Structure Analysis section, the author should further reveal the reasons for the short-term changes in systematic risk after Event 5. ③Are Figures 1 and 2 repeated twice? The display effect of the two figures are not clear. ④Besides different industries, do the nature of enterprises or the market value of companies also have different effects?***

We have fixed the event typo and made the correction. It is Event 10 which affects the food and drug retailers.

We have inserted an explanation in the second paragraph on page 23 to explain why short-term systematic risk changes after Event 5.

Figures 1 and 2 are different. Figure 1 shows the short-term changes in systematic risks while Figure 2 shows the long-term changes in systematic risks. The differences can be seen via the diamond risk structure. In the short-term, diamond risk structures appear in Event 5 and Event 9 while, in the long-term, diamond risk structures appear in Event 7. We have added an explanation for the diamond risk structure in the long-term in the first paragraph on page 24. Figures 1 and 2 have been changed to Figures 2 and 3.

The nature of enterprises or the market value of companies might have some effects. In this study, we examine the nature of the enterprises by estimating the abnormal returns using the market integration whereby some sectors might be highly integrated with the international stock markets in nature. Regarding the market value of companies, we have estimated the abnormal returns using the Fama-French 5 Factors model which controls for the size effects of the companies as a robustness test.

5. ***Conclusion: Introduction mentions the significance of studying the impact of EVFTA on the Vietnam stock market, which mentioned “It ... which will be useful to investors and policymakers in general.”The conclusions now only involve a summary of the empirical results. Policy implications need to be added to echo the previous views.***

We have added the first paragraph on page 26 to show the implications of our results.

**Reviewer #2:** The manuscript is interesting, although the topic is not new, at least from the perspective of investigating the effects of trade agreements on industries and stock markets. However, it is argued that one of the contributions of this manuscript resides in the analysis of these effects for a developing economy, Vietnam, that has recently signed a free-trade agreement with the EU. The manuscript needs several changes before being published.

***1. The Introduction should stress more the originality and contributions to the literature, particularly since the topic is not new.***

In response to the reviewers' suggestions, we have made significant revisions to the manuscript. Firstly, we have restructured the introduction section as follows: (1) Providing context on Vietnam and its Free Trade Agreements (FTAs), (2) Discussing the specific context of Vietnam in relation to the EU-Vietnam Free Trade Agreement (EVFTA) and its impact on the country, (3) Highlighting the gap in existing research and outlining the contributions of our study, (4) Explaining the rationale for examining the EVFTA and its implications for the Vietnam Stock market, and (5) Presenting an overview of the structure of our research.

We added a paragraph to emphasize more the originality and contributions to the literature (Paragraph 2 on page 4, paragraphs 2 and 3 on page 5).

***2. The Literature review needs organisation and structure. As it is, it looks amalgamated and constructed from various paragraphs that do not logically follow one after the other. Also, i encourage the authors to use the literature review to build their research questions and hypotheses, by highlighting the research gap.***

We have revised and restructured our literature and hypotheses development section by summarizing existing literature and providing empirical literature with targeted markets. We have provided discussions on the impact of FTA news and events on the stock market return, and on how the EVFTA news and events provide different impacts on various industry sectors in the Vietnam stock market. We have consolidated the discussion on the impact of FTAs on the market risk across industries and countries. In so doing, we have included the recent related literature, and thus highlighted the research gap from the existing literature and formulated the three testable hypotheses as follows.

H1: EVFTA-related events significantly affect the Vietnam stock market.

H2: The effects of EVFTA-related events vary across Vietnamese sectors.

H3: EVFTA announcements have different impacts on systematic risk across the sectors in Vietnam.

***3. The methodology is well explained, but the low number of events included in the study (10) might impact the findings***.

The events are collected and synthesised from various sources as indicated in Section 4. The events in the study are the major events and most relevant to EVFTA. Other events (if any) will be somewhat linked to these major events and the effects will be captured in our market anticipation and delayed reaction analysis whereby we analyse how the sectors reacted before and after these major events (see Tables 4 to 6).

***4. The results need to be better explained, particularly the differences between returns' reaction to each of the 10 events, as well as those between the economic sectors.***

We have added an explanation in paragraph 2 on page 19 to explain the difference differences between returns' reaction to event 6 and why the reactions are different between the sectors.

***5. There is no discussion on the results, the authors do not contrast their findings against other studies***

We have contrasted our findings to several studies in the current literature in paragraph 2 on page 19, paragraph 2 on page 20 and paragraph 1 on page 23.

***6. Conclusions need to highlight the implications of the findings, as well as to present the limitations of the study and the directions for future research that might be identified based on the results.***

We have highlighted the implications of the findings and the limitations in paragraphs 1 and 2 on page 26.
